# Generation of inner ear sensory neurons using blastocyst complementation in a *Neurog1*^+/−^−deficient mouse

**DOI:** 10.1186/s13287-021-02184-1

**Published:** 2021-02-12

**Authors:** Aleta R. Steevens, Matthew W. Griesbach, Yun You, James R. Dutton, Walter C. Low, Peter A. Santi

**Affiliations:** 1 Department of Ophthalmology, University of Minnesota, Minneapolis, MN USA; 2 Department of Neurosurgery, University of Minnesota, Minneapolis, MN USA; 3 Stem Cell Institute, University of Minnesota, Minneapolis, MN USA; 4 Department of Otolaryngology, University of Minnesota, Minneapolis, MN USA; 5 Mouse Genetics Laboratory, University of Minnesota, Minneapolis, MN USA

**Keywords:** Inner ear, Spiral ganglion neurons, Neurogenin1, Blastocyst complementation, Stem cells, Regenerative medicine

## Abstract

This research is the first to produce induced pluripotent stem cell-derived inner ear sensory neurons in the *Neurog1*^*+/−*^ heterozygote mouse using blastocyst complementation. Additionally, this approach corrected non-sensory deficits associated with *Neurog1* heterozygosity, indicating that complementation is specific to endogenous *Neurog1* function. This work validates the use of blastocyst complementation as a tool to create novel insight into the function of developmental genes and highlights blastocyst complementation as a potential platform for generating chimeric inner ear cell types that can be transplanted into damaged inner ears to improve hearing.

**Supplementary Information:**

The online version contains supplementary material available at 10.1186/s13287-021-02184-1.

## Introduction

Hearing loss is the most common neurosensory deficit. Approximately 488 million individuals world-wide and 15% of Americans have some degree of hearing loss [1]. Hearing depends on the mechano-sensory hair cells (HCs) and their innervating neurons, the spiral ganglion neurons (SGNs), which are responsible for transmitting auditory information from the HCs in the organ of Corti to the cochlear nucleus in the brainstem. Mammalian HCs and SGNs do not regenerate after damage, which results in sensorineural hearing loss (SNHL) [2]. In addition, auditory neuropathy with relative preservation of hair cells is a substantial cause of deafness [3, 4]. Cochlear implants are the only established therapy for severe to profound hearing loss; however, they require a viable SGN population for their success and efficacy [5].

Using exogenous stem cells to replace lost inner ear neurons is a potential strategy if stem cell-derived neurons can form central and peripheral connections, form synapses on hair cells and cochlear nucleus neurons, and re-establish functional and tonotopic circuits [6]. While, early attempts to target cochlear tissues using stem cells largely produced unremarkable results [6, 7], two promising in vivo studies have shown that stem cells can survive and supplement SGNs within a cochlea and even partially restore hearing function [8, 9]. However, there are two potential weakness in the previous studies: (1) since the transplanted stem cells were differentiated in vitro to form otic progenitors and due to the lack of using established markers of inner ear specific neurons, these cells may not be equivalent to SGNs formed in vivo, which may explain the observed limited functional recovery, and (2) using allogenic stem cells requires that the transplant recipient may need to undergo long-term immunosuppression to prevent rejection of the transplanted progenitors. We address these potential limitations by adopting the technique of blastocyst complementation (BC) to generate inner ear neurons from induced pluripotent stem cells (iPSCs).

BC is a technique in which deletion of a key gene for the development of a specific lineage creates a vacant niche (organogenesis-disabled phenotype) that can be complemented by the progeny of wild type pluripotent stem cells injected into embryos at the blastocyst stage of development. Resulting chimeras from BC have successfully generated entire, functional organs, such as the pancreas, kidney, eye, and lung, derived from the progeny of donor stem cells, by complementing genes necessary for their formation (pancreatic and duodenal homeobox 1 (PDX1), Sal-like like protein 1 (SALL1), melanocyte inducing transcription factor (MITF), and fibroblast growth factor receptor 2 (FGFR2), respectively [10–15].

These studies, using both intraspecies (mouse X mouse [11, 14] and pig X pig [12]) and interspecies (rat X mouse [10, 13]) chimeras, demonstrated the feasibility of BC to generate organs. Organogenesis is a complex developmental process requiring hierarchical cell and tissue differentiation, coordinated in time and space in response to changes in local and distant signaling cues. Replicating these conditions in vitro to generate functional tissues, let alone the organs, has proven extremely challenging and using the embryo to initiate the appropriate signaling cascades is a significant advantage of a BC approach. BC is the only current method for making fully functional, three-dimensional organs from pluripotent cells and generating human organs in large mammalian hosts may be able to address the critical worldwide problem of organ shortages for transplantation [16]. However, several hurdles still need to be overcome to make interspecies organ farming a reality [17]. For one, all components of an organ will need to be derived from the human tissue, which includes the endothelial, stromal, and possibly the immune cells [18–20]. While progress has been made on this front [21], it will take time to advance to point of being able to generate entirely the whole organs with BC.

By contrast, the replacement of specific cell types could improve health conditions such as pancreatic beta islet cells for type 1 diabetes [22, 23], dopamine-secreting neurons in Parkinson’s disease (PD) [24], and inner ear sensory cells for hearing disorders and may be a more readily attainable goal. BC has been used to generate rat islet cells that normalized glucose levels after transplantation into diabetic mice [25], and candidate genes have been identified to create replacement dopaminergic neurons using BC for PD [16]; however, BC has not been used to generate sensory cell types in the inner ear. Here, we present the first demonstration using intraspecies BC to create mouse donor stem cell-derived inner ear sensory neurons.

Ma and colleagues [26, 27] developed a *Neurogenin1* (*Neurog1*)^*−/−*^ knockout mouse that lacked an otic ganglion at embryonic day (E)10.5, which translated into postnatal day (P)0 inner ears that lacked afferent, efferent, and autonomic nerve fibers. In preliminary experiments using the *Neurog1*^−/−^ mutant mouse, we produced the first histological analysis of E18.5 *Neurog1*^*−/−*^ null inner ears [28], which confirmed the findings of Ma and colleagues. With the goal of generating stem cell-derived inner ear neurons, the *Neurog1*^*−/−*^ knockout animal was the optimal choice for creating a vacant niche to generate inner ear neurons by blastocyst complementation.

## Methods

All methods performed in this manuscript were in accordance with all policies of the University of Minnesota Institute of Animal Care Use Committee (IACUC), which approved the use and housing of these animals according to accepted principles of laboratory animal care (National Research Council 2003).

### Mice

The *Neurog1*^*tm1And/J*^ mouse strain was used [26, 27] (Jackson Laboratory, Stock No. 017306*)* in which the coding exon for *Neurog1* was replaced with a green fluorescent protein (GFP) cassette fused to a PGK-neo cassette successfully abolishing gene function in *Neurog1*^*−/−*^ null mice [26–28]. Expression of the GFP mRNA was reported to mirror *Neurog1* endogenous gene function which confirmed the specificity of the transgene knock in. However, GFP protein expression was undetectable indicating that the GFP transcript generated in these mice results in a nonfunctional GFP protein [26]. Mice were housed in a specific pathogen-free (SPF), Research Animal Resource (RAR) and American Association for Accreditation of Laboratory Animal Care, (AAALAC) approved facility, and plastic cages that were steam cleaned and autoclaved 3 times per week. The mouse colony was maintained by crossing *Neurog1*^*+/+*^ wildtype mice with *Neurog1*^*+/−*^ heterozygous mice. Experimental mice were generated through heterozygous matings (*Neurog1*^*+/−*^ X *Neurog1*^*+/−*^), which produced the following combinations of genotype: *Neurog1*^*+/+*^ wild-type*, Neurog1*^*+/−*^ heterozygous, *Neurog1*^*−/−*^ null mice. *Neurog1*^*−/−*^ null mice die 24 h after birth due to their inability to suckle and were only generated for blastocyst complementation or embryonic harvest. Toe clips were collected at 1 week of age, which were subsequently hydrolyzed for genotyping analysis. Genotyping was performed by polymerase chain reaction (PCR) using the Jackson Laboratory’s validated genotyping protocol: https://www.jax.org/Protocol?stockNumber=017306&protocolID=23926.

Genotyping was performed with the following primers: WT forward (ACCACTAGGCCTTTGTAAGG), mutant forward (ATAGACCGAGGGCAGCTTCA), and a common reverse primer (CGCTTCCTCGTGCTTTACGGTAT). Genotyping was run in two separate reactions: (1) WT forward, a sequence which detects endogenous Neurog1 protein, and the common primer and (2) the mutant forward, which is a sequence in the neomycin cassette, and the Common primer. This reaction yields a 198-bp wild-type band and a 500-bp mutant band. Wild-type animals will have only one wild-type band, heterozygotes will have both wild type and mutant bands, and homozygous nulls will only have a mutant band (Supplemental Figure 1).

### Blastocyst complementation

Mouse x mouse blastocyst complementation was performed by injecting membrane-bound GFP-labeled mouse iPSCs into *Neurog1-*deficient blastocysts. The derivation of the 3F10 iPSC line used was previously described in detail by Greder and colleagues [29]. Briefly, the iPSCs were derived from Oct4-MerCreMer mTmG mice. These mice are from a cross between Oct4-MerCreMer mice carrying a tamoxifen-inducible Cre knock-in transgene upstream of the 3′UTR of Oct4 (Jackson Laboratory, Stock No: 016829) and a homozygous tdTomato/EGFP reporter mouse strain (mTmG) (Jackson Laboratory, Stock No. 007576). iPSCs were generated according to previous reprogramming protocols [30, 31] and resulting pluripotent iPSCs were cultured with tamoxifen diluted in miPSC growth medium to 100 nM. This strategy results in all donor iPSCs being irreversibly labeled with membrane-bound EGFP (Supplemental Figure 2). Injected blastocysts were transferred to pseudopregnant female surrogates, where they were allowed to develop until the time of natural birth. To produce mutant blastocysts, an in vitro approach was used. To do this, the egg donors (*Neurog1*^*+/−*^ heterozygous female mice) were superovulated [32] at 3–4 weeks of age, by giving CARD HyperOva® (Cosmo Bio, Cat. No. KYD-010-EX-X5) 0.1 ml/mouse, intraperitoneally (*i.p*.), at 17:30 pm (mouse room light:dark cycle: 6:00–20:00), followed by human chorionic gonadotropin (HCG) (Sigma-Aldrich Cat. No. C 1063), 5 insulin units (IU)/mouse 47–48 h later. Fresh sperm from a *Neurog1* heterozygous (+/−) male was used for IVF. Fertilized eggs were cultured in home-made modified human tubal fluid (mHTF, a.k.a. high calcium HTF) medium until the blastocysts were formed in ~ 72 h after IVF and ready for microinjection of iPSCs. Each blastocyst was injected with 10–15 iPSCs. After blastocyst injection, mouse blastocysts were transferred into the uteri of pseudopregnant surrogate mice. This entire process was performed on three separate occasions. Variability is expected with embryological development and some of the surrogate dams resorbed all the developing embryos. However, we obtained two viable litters and 13 P0 pups were obtained: 2 *Neurog1*^*+/+*^ wild type animals, 9 *Neurog1*^*+/−*^ heterozygote animals, and 2 *Neurog1*^*−/−*^ homozygote null animals. Of these, one of the wild type samples was highly chimeric, three of the heterozygotes were complemented (30%), and none of the homozygotes exhibited any stem cell-derived chimerism.

### Inner ear harvesting from the embryo, cryosectioning, and immunohistochemistry

For tissue harvesting, P1 mice were euthanized using C0_2,_ according to RAR guidelines, followed by decapitation. The heads were bisected with the intention of using one inner ear for cryosectioning and immunohistochemistry (IHC) and the other processed for imaging using scanning Thin Sheet Laser Image Microscope (sTSLIM). The bisected heads were fixed overnight in 4% paraformaldehyde (PFA) in phosphate-buffered saline (PBS) and were rinsed with PBS twice for 15 min each before undergoing decalcification in 10% ethylenediaminetetraacetic acid (EDTA) for 3 days. Inner ears to be analyzed by cryosectioning and IHC were cryoprotected through overnight incubations in ascending concentrations of sucrose up to 30% and embedded in tissue-freezing medium (TFM) (General Data, Cat#: TFM-5) and snap-frozen on dry ice. Bisected heads containing the P0 inner ears were sectioned on a cryostat at a thickness of 16 μm and directly mounted to slides. The entire ear was collected, starting with sections from the cochlea, through the macular organs, and through the cristae and semicircular canals. Slides were allowed to dry on the slide warmer for a minimum of 1 h prior to beginning staining or storing at − 20 °C. The sectioned tissue was always stained within 3 days of sectioning. If previously frozen, the tissue was rewarmed to room temp by placing it on the slide warmer for a minimum of a half hour. Prior to performing immunohistochemistry, epitopes were exposed by performing antigen retrieval. Briefly, slides were placed in Coplin jars filled with boiling sodium citrate buffer with Tween® 20 (Sigma-Aldrich Cat# P9416) (pH adjusted to 6.0 with 1 N hydrogen chloride (HCl)) and incubated in a steamer for 20 min. Slides were allowed to cool to room temperature, for at least an hour, before continuing the staining protocol. After antigen retrieval, the slides were dried and the sections were outlined with a hydrophobic barrier pen (Super^HT^ Pap Pen, Polysciences Cat# 24230-1). The tissue was rinsed twice (10 min each rinse) with PBS and rinsed twice (15 min each rinse) in PBS with 0.1% Triton™ X-100 (Sigma-Aldrich, X100, Cat# 9002-93-1) (PBST). Non-specific binding was blocked against using 10% normal horse serum (ThermoFisher, Cat# 16050122) in 0.1% PBST for 1 h at room temperature. Primary antibodies, diluted in the blocking solution, were applied and allowed to incubate in the 4 °C cold room overnight. All antibodies are listed in the below table (Tables 1 and 2). The following day, the slides were rinsed with four 15-min washes in 0.1% PBST, and sections were incubated in secondary antibodies, diluted in blocking solution, for 2 h at room temperature. The tissue was then rinsed in 0.1% PBST with two 10-min washes and counterstained with 4′,6-diamidino-2-phenylindole (DAPI, Thermo Fisher Scientific, Cat# D1306) at a concentration of 1:5000 for 5 min. Lastly, the tissue was rinsed for 10 min in PBS, and coverslips were mounted to the slide after applying the mounting medium with anti-fade agent (Electron Microscopy Services, Cat# 17985-11). Slides were sealed using CoverGrip™ Coverslip Sealant (Thermo Fischer Scientific, Cat# NC0154994).

**Table 1:** Antibodies

Antibody	Vendor	Cat#	Dilution
rabbit polyclonal α-MYO6	Proteus Biosciences Inc.	25-6791	1:500
chicken α-GFP	Abcam Inc.	ab13970	1:1000
mouse monoclonal α-Tuj1	Biolegend	MMS-435P	1:1000

**Table 2:** Antibodies

Antibody	Vendor	Cat#	Dilution
488 donkey α-chicken	Jackson ImmunoResearch Alexa Fluor	703-545-155	1:1000
555 donkey α-rabbit	Invitrogen Alexa Fluor	Ab150062	1:1000
647 donkey α-mouse	Invitrogen Alexa Fluor	A-31571	1:1000

### Microscopy and image processing

All immunohistochemical imaging was performed on a Leica inverted light microscope. Images were exported as raw Leica Image File Format (LIF) files and processed in FIJI (Fiji Is Just ImageJ). The resolution of each image was adjusted to 300 dots per inch, which reflects pixels per inch (DPI) in Photoshop, and all resulting JPEGs were assembled using Adobe Illustrator.

### sTSLIM macro light-sheet microscope

In 2008, we developed a high-resolution microtome/microscope called scanning Thin Sheet Laser Image Microscope (sTSLIM) that can image whole cochleas, nondestructively [33–37]. sTSLIM optically sections and digitizes all cochlear tissues to allow for a complete quantitative assessment of normal and pathological structures. The Santi laboratory has used sTSLIM to characterize mouse cochlear development [38], analyze normal spiral ganglion cell number in the mouse [39], and illustrate alterations in cochlear structures in two knock-out mouse models (Atonal homolog1, ATOH1 and *N*-Myc proto-oncogene protein, *N*-Myc) [40, 41]. Since light scatter and absorption are the greatest limiting factors in resolution, we have performed both tissue engineering (improved transparency and accessibility to antibody labeling through decellularization) and optical engineering (scanned light-sheet, Bessel beam illumination, structured illumination, confocal line detection, and radial sectioning) to improve imaging of large specimens such as the mouse cochlea with portions of the brain attached. Microscope performance will be tested on a regular basis using 150-nm gold fluorescent beads to ensure that the point spread function of the microscope is stable and optimal for reproducible imaging.

### Tissue preparation for sTSLIM

Inner ears processed for sTSLIM were fixed and decalcified (described above) and then underwent a dehydration series in ascending concentrations of ethanol (30%, 50%, 70%, 100% EtOH) and then lightly stained by rhodamine-B isothiocyante (5 μg/mL in 100%) for 1 h. After rinsing in 100% EtOH, inner ears were cleared to transparency with BABB (benzyl alcohol: benzyl benzoate 1:2), and specimens were mounted to a specimen rod and placed in a BABB-filled chamber for imaging by sTSLIM.

### sTSLIM imaging

sTSLIM optically sections non-destructively by moving a thin light sheet in the *x*- and *Z*-axes. A *z*-stack of well-aligned 2D optical sections of the inner ear was automatically imaged with *x*-axis scanning across the width of the specimen and with a *z*-step size of 5 μm. At this thickness, the whole mouse cochleae contained ~ 300 images that take ~ 30 min to produce. Images were adjusted for brightness, contrast, and either unsharp masking or deconvolution using ImageJ (National Institutes of Health). The *z*-stack was then loaded into the Amira 3D program, and structures of interest were manually segmented, by drawing along their borders in different colors to prepare 3D reconstructions and volume calculations. Supplemental Figure 3 shows an example of a 2D optical section through the cochlea and Supplemental Movie 1 shows segmented inner ears rotating horizontally.

## Results

### Donor GFP-labeled stem cells create chimeric spiral ganglion and vestibular neurons in the *Neurog1*^*+/−*^ heterozygote

**Fig. 1:**
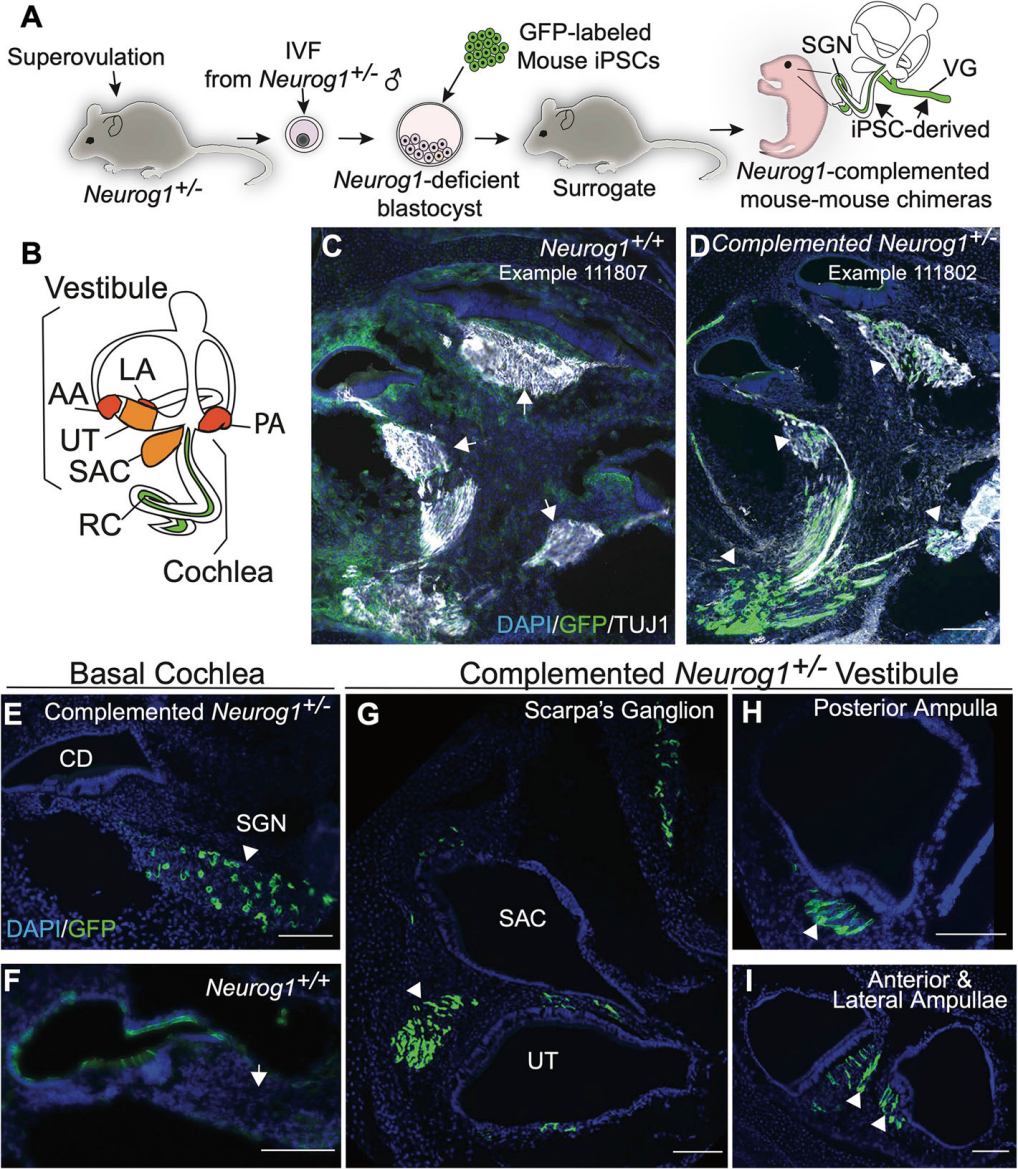
Donor GFP-labeled stem cells create chimeric spiral ganglion and vestibular neurons in the *Neurog1* heterozygote. **a** Schematic of experimental methods for BC. *Neurog1*^*+/−*^ female mice were superoverulated with hormones. Zygotes were extracted and fertilized by in vitro fertilization (IVF) with *Neurog1*^*+/−*^ male sperm and allowed to mature until blastocyst stage (~E3.5), at which point all blastocysts were injected with GFP-labeled iPSCs. Complemented blastocysts were transferred to a pseudopregnant surrogate. Pregnancies were to taken to birth, embryos were harvested, and complementation in the inner ear was assessed. **b** Diagram of the inner ear indicates the vestibular (balance) portion and cochlear (auditory) portion. Low magnification of the midmodiolar region of the cochlea in **c** representative image of the *Neurog1*^*+/+*^ wild type inner ear shows the absence of GFP-labeled stem cells in the SGN (arrows) (images are from a single *Neurog1*^*+/+*^ wild type (1/2), sample number 111807) in contrast to **d** specific stem cell complementation in the SGN in a *Neurog1*^*+/−*^ heterozygote (arrowheads) (images are from a single complemented *Neurog1*^*+/−*^ heterozygote (1/3), sample number 111802). High-magnification images of the basal cochlear turn show **e** the presence of GFP-labeled stem cells in a *Neurog1*^*+/−*^ heterozygote SGN (arrowhead) and absence in **f** the *Neurog1*^*+/+*^ wild type SGN (arrow). Specific stem cell complementation was seen in the vestibule of a *Neurog1*^*+/−*^ heterozygote, **g** in Scarpa’s ganglion adjacent to the utricle (UT) and saccule (Sac), **h** neurites (arrowhead) innervating the posterior ampulla, and **i** neurites innervating the anterior and lateral ampullae (arrowheads). AA anterior ampulla, LA lateral ampulla, PA posterior ampulla, Ut utricle, SAC saccule, RC Rosenthal’s canal with SGNs. All scale bars are 100 μm. Scale bar in **d** also corresponds to **c**

*Neurog1*-deficient blastocysts were established by performing in vitro fertilization (IVF) with zygotes extracted from *Neurog1*^*+/−*^ heterozygous dams and fresh sperm from *Neurog1*^*+/−*^ heterozygous male mice. Blastocysts were injected with EGFP-labeled mouse induced pluripotent stem cells (iPSCs) at approximately embryonic day (E)3.5 and subsequently were transferred into surrogate pseudopregnant dams (Fig. 1a). The iPSCs were derived from transgenic mice that carried a tamoxifen-inducible CreER transgene under the control of the *Oct4* promoter and a Cre-dependent tdTomato/EGFP reporter cassette. The iPSCs used in this study were from cells previously cultured with tamoxifen to activate the Oct4 driven CreER, removing the tdTomato reporter gene and activating EGFP expression. As Oct4 is highly expressed in iPSCs, this strategy irreversibly labels all the donor stem cells and their progeny with EGFP (Supplemental Figure 2). Upon analysis of chimeric inner ears at P1, robust and specific incorporation of GFP-labeled cells derived from the donor stem cells was seen in the SGN of a *Neurog1*^*+/−*^ heterozygote (Fig. 1).

In the complemented *Neurog1*^*+/−*^, GFP-labeled iPSCs contributed to the SGN and descending neuronal processes in the cochlea with minimal donor stem cell-derived cell incorporation in other tissue (Fig. 1d, e arrowheads). The specificity of labeling in the *Neurog1*^*+/−*^ was unmistakable, given the lack of GFP labeling observed in the wild type SGN (Fig. 1c, f, arrows). Progeny of the GFP-labeled donor iPSCs also contributed to the cell bodies of Scarpa’s ganglion (Fig. 1g), in addition to vestibular neurons innervating the cristae ampullaris (Fig. 1h, i). Since *Neurog1* is known to be required for the formation of both cochlear and vestibular neurons [27], these results indicate that the integration pattern of cells derived from the wild type donor cells recapitulates that expected from cells with wild type endogenous *Neurog1* gene function and that *Neurog1* haplodeficiency creates a vacant niche that can be filled by cells derived from exogenous stem cells to produce SGNs using BC.

### Complementation of *Neurog1*-deficiency is distinct from general chimerism

A chimera is defined as a composite animal comprised of two genetically distinct cell populations [42]. Performing BC in wild type animals will result in random chimerization throughout the developing embryo [43, 44]. When performing BC in knockout blastocysts, in addition to exogenous donor-derived cells targeting a vacant developmental niche, random chimerism can also be observed in non-targeted cell types.

**Fig. 2:**
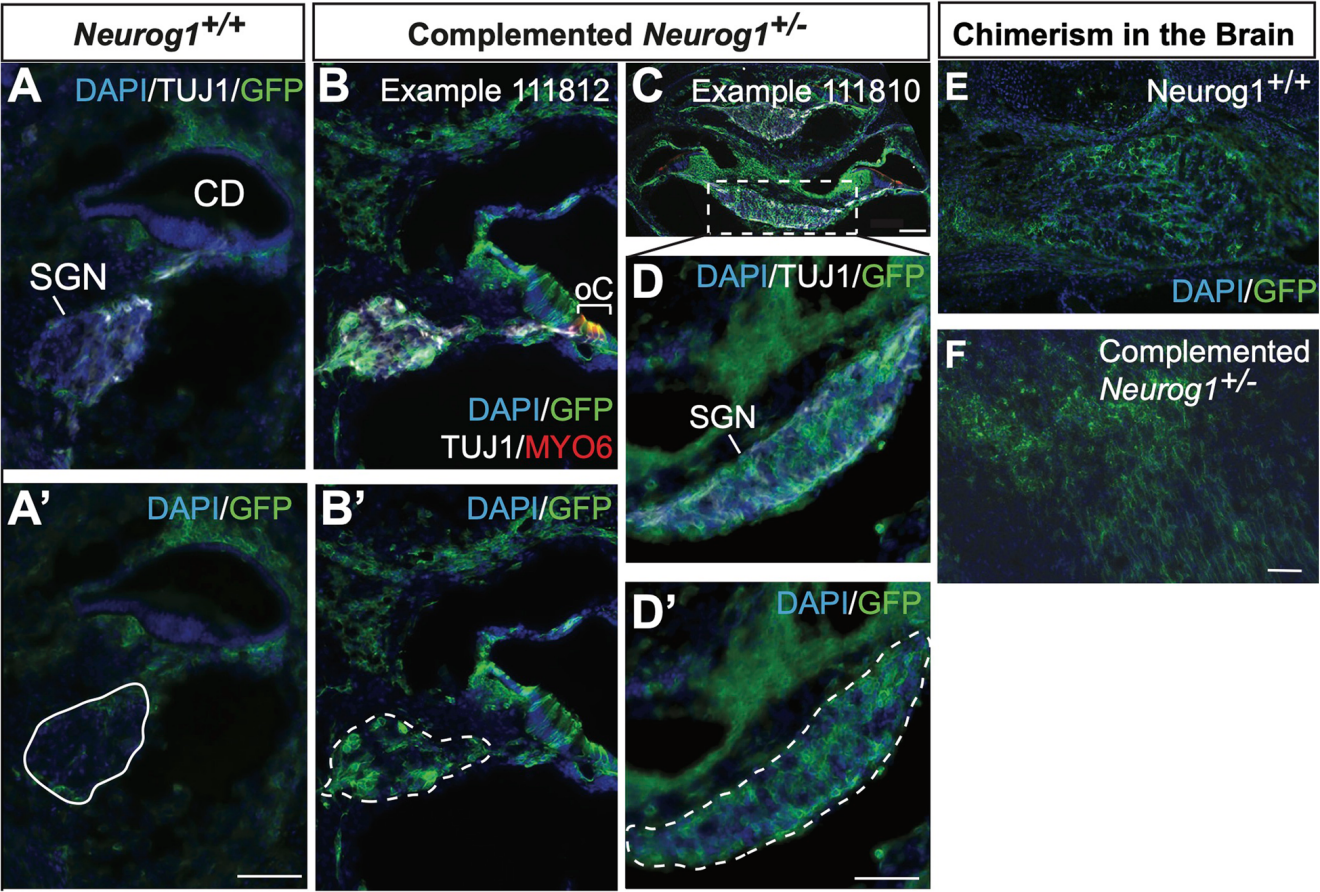
Complementation of *Neurog1*-deficiency is distinct from general chimerism. **a**, **A’** High magnification of the basal turn in a chimeric *Neurog1*^*+/+*^cochlea does not show the engraftment of iPSCs in the spiral ganglia, (solid outline) compared to **b**–**D’** examples from two separate chimeric and complemented *Neurog1*^*+/−*^ cochleas in which iPSCs extensively contribute to the SGN (dotted outline). Chimerism was seen in both the **e** *Neurog1*^*+/+*^ brain and **f** the *Neurog1*^*+/−*^ brain*,* indicating that while both embryos successfully formed chimeras, donor cells did not specifically contribute to the SGN in the absence of a vacant niche. (Images are from a single *Neurog1*^*+/+*^ wild type (1/2), sample number 111807 and two separate complemented heterozygotes (2/3) sample numbers 111812 and 111810). All scale bars are 100 μm. Scale bar in **A**’ corresponds to **a**–**B’**, scale bar in **f** corresponds to **e**. DAPI 4,6-diamidino-2-phenylindole, TUJ1 class III beta-tubulin antibody, GFP green fluorescent protein antibody against endogenous fluorescence, MYO6 unconventional myosin VI antibody

Two examples of complemented *Neurog1*^+/−^ heterozygotes were highly chimeric, as a high degree of specific donor-derived cell integration into the SGN was observed (Fig. 2b–D’), in addition to general chimerism, which was evident from the widespread GFP expression in non-sensory otic cell types (Fig. 2b–D’). This general chimerism is similar to that observed in the *Neurog1*^*+/+*^ control, which also displayed the incorporation of GFP-expressing cells in non-sensory cells. Notably, the *Neurog1*^+/−^ heterozygote SGNs, in some regions, appeared to be derived nearly entirely from donor iPSCs, whereas the *Neurog1*^*+/+*^ wild type SGNs were consistently negative for GFP expression (Figs. 1c and 2a, A’).

The extent of donor-derived cell chimerism in the *Neurog1*^*+/+*^ wild type in comparison to the complemented *Neurog1*^*+/−*^ heterozygotes was assessed further by looking at the respective level of GFP expression in the temporal lobe of the brain. This analysis clearly showed the incorporation of exogenous iPSC-derived cells in the *Neurog1*^*+/+*^ brain at a comparable level to the complemented *Neurog1*^*+/−*^ heterozygotes (Fig. 2e, f). Therefore, chimeras successfully formed regardless of genotype, but in the absence of a *Neurog1-*deficient niche, GFP-expressing cells derived from the exogenous donor iPSCs could not contribute to the formation of the SGN in wild type inner ears (Figs. 1f and 2Aa’)*.* These results support our hypothesis by demonstrating that BC can specifically generate SGNs using WT donor stem cells.

We did not see complementation in the two *Neurog1*^*−/−*^ mutant embryos that we obtained. However, both nulls showed few to no GFP-expressing cells engrafted throughout the whole embryo, suggesting that the lack of complementation was due to low chimerism*.* With an increased sample size*,* we expect to obtain highly chimeric *Neurog1*^*−/−*^ mutants in which all of the SGNs are derived from donor stem cell progeny. Therefore, it is anticipated that, following Mendel’s law, 75% of blastocysts (heterozygous and null) obtained from BC will give rise to chimeric inner ears.

### Donor stem cells extensively contribute to the complemented *Neurog1*^+/−^ vestibule

**Fig. 3:**
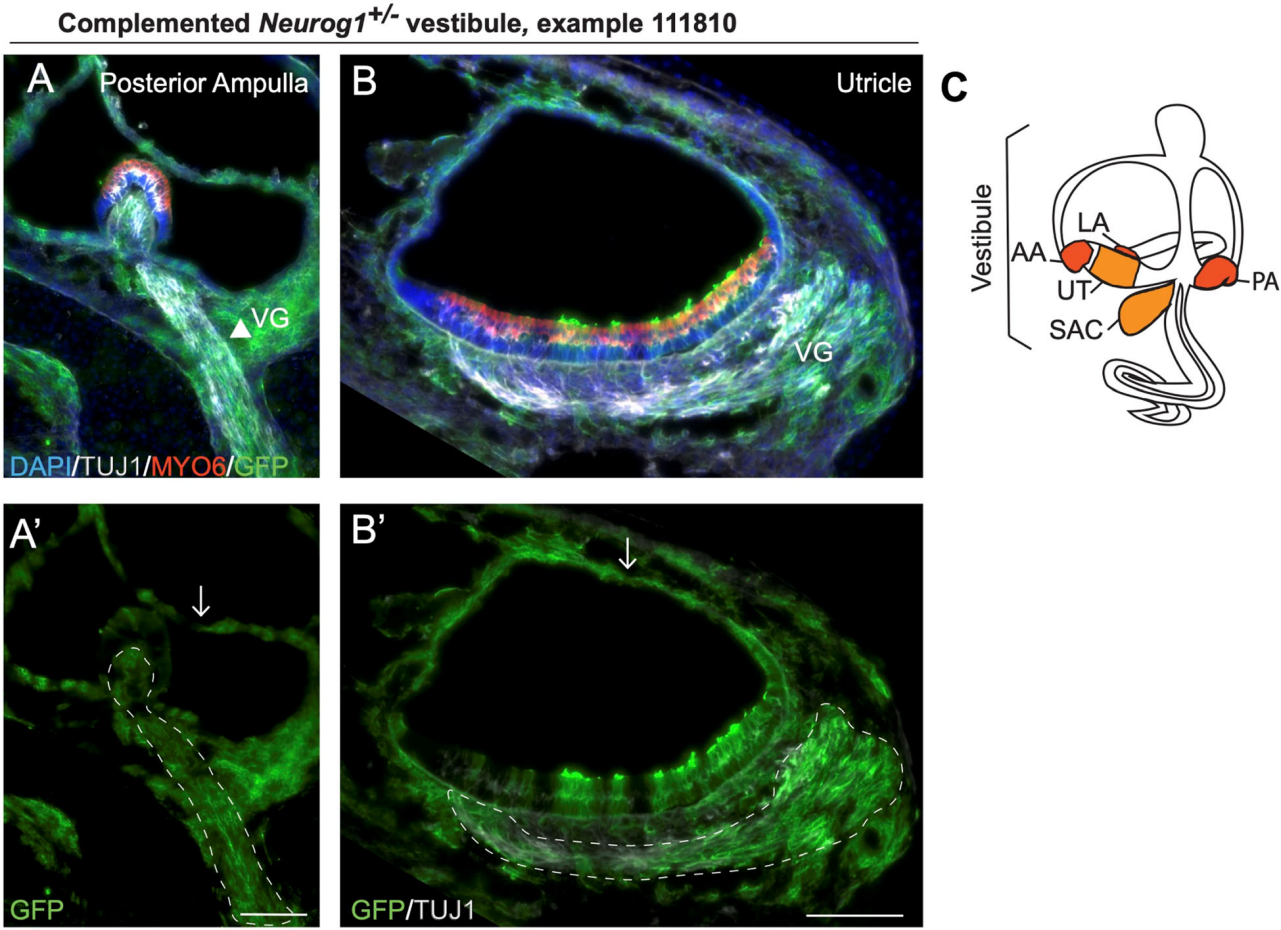
Donor stem cells extensively contibute to a complemented *Neurog1*^+/−^ vestibule. High magnification of the (**a**, **A’**) posterior ampulla and (**b**, **B’**) utricle in a highly chimeric complemented *Neurog1*^*+/−*^ vestibule shows that GFP-labeled donor iPSCs contribute to the near entirely of both structures (*n* = 1, sample number 111810). GFP co-expresses with TUJ1-labeled vestibular nerves in the vestibular ganglia (VG) (dotted outlines), some MYO6-labeled vestibular hair cells (red), and non-sensory vestibular tissue (white arrows). **c** Inner ear diagram shows the anatomy of the vestibule with anterior ampulla (AA), lateral ampulla (LA), posterior ampulla (PA), saccule (SAC), and utricle (UT). VG vestibular ganglion. All scales bars are 100 μm

In the highly chimeric complemented *Neurog1*^*+/−*^ inner ears, the contribution of donor-derived GFP-labeled cells appeared to increase in sections through the more basal cochlea in a trend that was dramatically more evident in the vestibule. In fact, it appeared that the majority of cells in the vestibule were derived from donor iPSCs, given the extensive presence of GFP in all vestibular cell types. Specifically, GFP entirely co-expressed with the neuronal marker class III beta-tubulin (TUJ1) in the neurites innervating the vestibular sensory organs (Fig. 3a–B’, arrowhead and dotted lines), in addition to many Myosin 6 (MYO6)-expressing vestibular hair cells (Fig. 3a, b, red) and non-sensory cells (Fig. 3A’, B′, small arrows). While the degree of donor cell contribution to the complemented *Neurog1+/−* vestibule was striking, the biological significance of this was lacking, until a clear heterozygote effect in non-complemented *Neurog1*^*+/−*^ inner ears was detected.

### Blastocyst complementation rescues *Neurog1*^*+/−*^ inner ear malformations

**Fig. 4:**
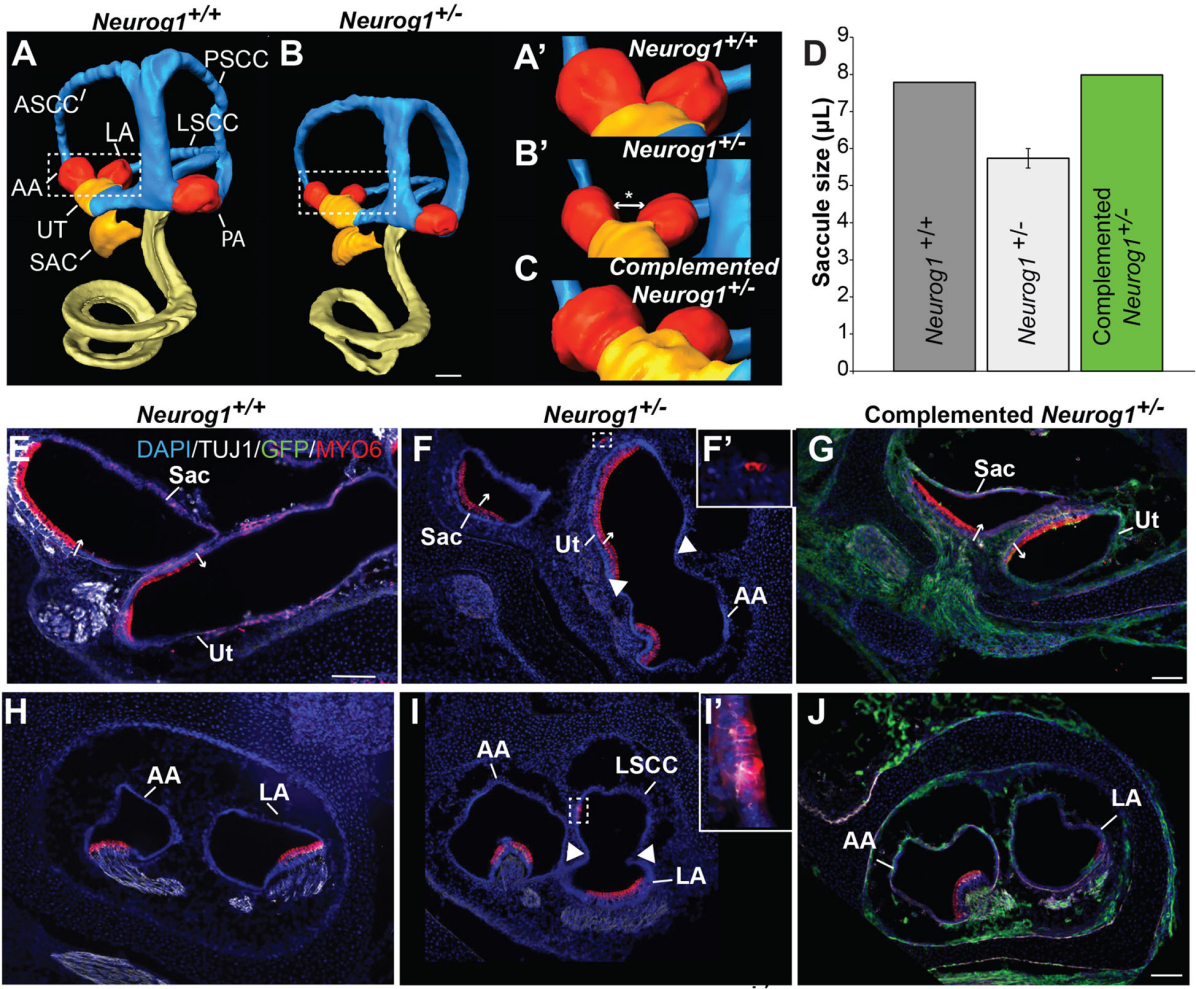
The *Neurog1*^*+/−*^ heterozygous vestibule showed non-sensory defects that were rescued by stem cell complementation*.* Three-dimensional reconstructions of **a** a *Neurog1*^*+/+*^ wild type inner ear compared to **b** a *Neurog1*^*+/−*^ inner ear, reveals a marked size and morphological difference between genotypes, which is particularly noticeable in the anterior (AA) and lateral ampullae (LA) (dotted boxes). **A’**,**B’** Higher magnification images of the dotted boxes from **a** and **b** and **c** a comparable region from a complemented *Neurog1*^*+/−*^ heterozygote, shows a rescue of vestibular morphogenesis. **d** Quantification of utricular volume in a *Neurog1*^*+/+*^ (*n* = 2), non-complemented *Neurog1*^*+/−*^ (*n* = 3), and a complemented *Neurog1*^*+/−*^ heterozygote (*n* = 1) shows the rescue of the non-complemented *Neurog1*^*+/−*^ utricular size with donor stem cell complementation. **e** Cross-section through the saccule (SAC) and utricle (UT) in a *Neurog1*^+/+^ control shows the characteristic orthogonal orientation of the sensory maculae that is reflected in the perpendicular orientation of the sensory hair cells in each organ (small arrows). By contrast, **f** the *Neurog1*^*+/−*^ heterozygous vestibule had a smaller saccule (SAC) and utricle (UT) and the hair cells in the sensory maculae were orientated in the same direction (small arrows, red). Additionally, the *Neurog1*^*+/−*^ heterozygous anterior ampulla (AA) was unusually close to the utricle (arrowheads) (*n* = 4). **F’** Ectopic hair cells were occasionally seen in the *Neurog1*^*+/−*^ heterozygous vestibular ganglia adjacent to the vestibule. These non-sensory and sensory defects were rescued in **g** complemented *Neurog1*^*+/−*^ heterozygote samples (*n* = 3). Similarly, compared to **h** the anterior (AA) and lateral ampullae (LA) in a *Neurog1*^+/+^ control, **i** the non-complemented *Neurog1+/−* heterozygous anterior ampullae (AA) was smaller and appeared connected to the lateral semi-circular canal (LSCC, arrowheads). **I’** Ectopic hair cells were sometimes seen in the semicircular canals (box). These defects were rescued in **j** complemented *Neurog1*^*+/−*^ samples. All scale bars are 100 μm. Scale bar in **b** also corresponds to **a**; scale bar in **g** corresponds to **e** and **f**; scale bar in **j** corresponds to **h** and **i**

Non-complemented *Neurog1*^*+/−*^ heterozygote inner ears were observed to have inner ear morphological non-sensory malformations, which included inner ears reduced in size by approximately 60% of the wildtype control (Fig. 4a, Supplemental Movie 1). Three-dimensional reconstructions revealed overt malformations particularly in the vestibule, in which the anterior and lateral ampullae and the saccule were notably reduced in size (Fig. 4a–d, Supplemental Movie 1). This finding was confirmed via cryosections, which displayed that the non-complemented *Neurog1*^*+/−*^ vestibule sometimes had an abnormally orientated saccule, utricle, and anterior ampullae. Specifically, unlike the typical orthogonal arrangement of the utricular and saccular maculae observed in the wild type vestibule, the non-complemented *Neurog1*^*+/−*^ sensory maculae (denoted by MYO6-expressing hair cells) were oriented in parallel (Fig. 4e, f; red, small arrows). Additionally, the utricular maculae and anterior crista ampullaris were unusually close to one another (Fig. 4f, arrowheads). Moreover, in some cases, the lateral ampulla appeared connected to the lateral semicircular canal (Fig. 4i, arrowheads). Impaired non-sensory vestibular morphology was observed in four of the six *Neurog1*^*+/−*^ heterozygotes obtained and analyzed. Strikingly, the remaining two *Neurog1*^*+/−*^ heterozygotes completely lacked a vestibule (not shown). Together these observations suggest that a reduction in non-sensory cell formation and/or a failure of sensory organ separation occurred with the reduction of *Neurog1* gene dose.

Cochlear and vestibular HCs developed normally in the *Neurog1*^*+/−*^ heterozygote (as has been reported for the *Neurog1*^*−/−*^ mutant) despite impaired non-sensory formation. However, sensory development potentially occurred at the expense of non-sensory development, as ectopic HCs were sometimes seen in non-sensory regions in the lateral semicircular canal (Fig. 4I’).

Importantly, non-sensory defects observed in the *Neurog1*^*+/−*^ heterozygotes (Fig. 4B, B', D, F, I)  were rescued in complemented *Neurog1*^*+/−*^ samples (Fig. 4C, D, G, J). Given the extensive contribution of GFP to non-sensory tissue in the complemented *Neurog1*^*+/−*^ heterozygous vestibular sensory organs (Fig. 3), these results suggest that widespread incorporation of cells derived from the donor iPSCs to the vestibule in the complemented *Neurog1*^*+/−*^ heterozygote is not random, but rather reflects the recovery of a previously unappreciated biological function of *Neurog1* in inner ear morphogenesis. This finding demonstrates the use of BC as a tool to elucidate novel gene function and to confirm or disprove concepts regarding the development of neurobiological systems.

## Discussion

Presented here for the first time is the intraspecies complementation of *Neurog1-*deficient mouse blastocysts to generate a chimeric population of donor iPSC-derived SGNs. This is a major advancement in the field of regenerative medicine for hearing disorders. The complexity of the intersecting developmental pathways involved in lineage specification of inner ear sensory cells is a difficult task to recapitulate in vitro [6]. By contrast, following blastocyst complementation, the developing embryo initiates these processes to produce what are presumed to be authentic spiral ganglion neurons. The approach of using BC to target *Neurog1-*deficiency is also advantageous, as *Neurog1* is well-characterized to specify inner ear neuroblasts during the otocyst stage, a developmental stage when the future inner ear is a simple structure on a cellular level [45–47]. Therefore, it would be a straightforward workflow to (1) use blastocyst complementation to target a *Neurog1-*deficient niche in order to generate chimeric otocysts that can (2) be surgically extracted, (3) GFP-labeled neuroblasts derived from donor cells isolated by flow cytometry, and (4) could be transplanted to inner ears with damaged neuronal innervation. The prospect of isolating and transplanting autologous BC-derived cells has been successfully demonstrated when pancreatic islet cells were extracted from mouse-rat chimeras and transplanted into a chemically induced mouse diabetes model and normalized host blood glucose levels for over a year [25]. Moreover, developing the therapeutic application for using BC to create inner ear neurosensory cells is simpler than the goal of using BC for the generation of entire organs, which may require the nullification of several genes, such as those required for the development of the vasculature system [48] in order to prevent immune rejection.

Many outstanding hurdles exist in the BC field, which includes matching the developmental timing between the host blastocyst and donor cells of different species, which is a particular challenge for creating chimeras with human tissue. However, great strides have been made in better understanding the optimal cellular status in PSCs (naïve vs primed) necessary for successfully creating interspecies chimeras, in addition to ways of promoting survival of the donor cells [49–54]. Therefore, these barriers seem likely to be overcome, as indicated by the creation of successful human-animal chimeras [55, 56].

Using iPSCs for BC was designed to circumvent ethical issues associated with using embryonic stem cells (ESCs) and with the goal of creating autologous organs/tissues for transplantation to avoid immune rejection by the recipient. While a number of studies support this conclusion by showing that transplanted derivatives of pluripotent stem cells do not evoke an immune response [57, 58], a few recent studies have raised concerns [59] that include potential immunogenicity of iPSCs. Specifically, it has been found that human-induced pluripotent stem cells (hiPSCs) can sometimes be rejected by allogeneic and autologous natural killer cells (NK) [60] and cells derived from iPSCs can similarly activate the immune system [19]. Due to these mixed results, caution will be warranted when moving interspecies BC into the transplantation arena. However, safer alternatives to immunosuppressants are being developed. Since rejection is primarily mediated by T cell-dependent recognition of foreign antigens [61], strategies such as modulation of the T cell costimulatory and inhibitory pathways are being developed to safely protect the iPSC-derived transplants from immune rejection [62, 63]. Other approaches to providing immune protection for transplants are by targeted disruption of the activation of antigen-presenting cells. For instance, blocking the expression of the major histocompatibility complex (MHC) class II transactivator in human embryonic stem cells (hESCs) leads to the silencing of HLA class II expression and thereby making these cells immune evasive. Therefore, there will likely be feasible options for developing safe and effective strategies to support iPSC-based regenerative medicine in the near future.

Unlike other studies using BC, we demonstrated that simply being haplodeficient for a master fate-determining gene is sufficient to generate a BC-derived tissue, which adds nuance to the complementation field. This unexpected finding led to the analysis of heterozygous inner ears with and without complementation and generated data that suggests that *Neurog1* is dose-dependently required for non-sensory development while largely preserving neurosensory development. This may suggest that *Neurog1* is involved in a binary decision to either adopt a non-sensory or neurosensory fate through an interaction with Notch signaling, a notion consistent with previous studies [26, 45, 46, 64]. Importantly, the observed non-sensory defects in the *Neurog1*^*+/−*^ were rescued in successfully complemented chimeric samples (Fig. 4). This supports the idea that our complementation results cannot be attributable to general chimerism, but rather are specific to an endogenous role for *Neurog1* in inner ear morphogenesis in addition to neurogenesis. *Neurog1* has been previously fate mapped in non-sensory regions of the cochlea and maculae [46]; therefore, this work provides evidence of a functional role for *Neurog1* in these areas and supports the likelihood of *Neurog1* playing additional roles in inner ear development beyond simply promoting inner ear neurogenesis.

Here, we establish, using intraspecies mouse chimeras, that BC is a platform that can be used for generating inner ear cell types. The generation of hiPSCs enables the generation of tissues that are either autologous or from closely related genetic background as the recipient [65]. Therefore, this work sets the stage for interspecies complementation with the goal of generating human inner ear tissues using swine as biological incubators, which could lead to improved research models and drug screening methodologies and ultimately transplantation to deaf patients to improve hearing.

## Supplementary Information

**Additional file 1: Supplemental Figure 1** Representative gel images of genotyping for a litter from the *Neurog1* colony. Top gel is the mutant genotyping, and the bottom gel is the wild type genotyping for the same samples. Wild type (lane 1): 198 bp. Heterozygote (lanes 2-4,6-13): 198 bp and ~ 500 bp. Mutant (lane 5): ~ 500 bp. Heterozygote positive controls (lanes 11-13) were always run in triplicate due to occasional variability in their result. All genotyping was run 3 times for each sample to confirm genotype. **Supplemental Figure 2.** Representative examples of GFP-expressing induced pluripotent stem cell (iPSC) colonies. (A) GFP expression. (B) Phase Contrast. Note that all of the iPSCs are GFP-labeled. Scale Bar: 100 μm. **Supplemental Figure 3** 2D optical section through a wild type cochlea using sTSLIM. Bracket indicates the sensory cells of the organ of Corti. Note the high degree of resolution throughout the image, in particular the cell bodies in the SGN. Scale Bar: 100 μm. oC: organ of Corti; SGN: Spiral ganglion neurons.

**Additional file 2: Supplemental Movie 1.** An 8 s video showing segmented and three-dimensionally reconstructed *Neurog1*^*+/+*^ and *Neurog1*^*+/−*^ inner ears rotating horizontally, to highlight the morphological differences observed between wild type and non-complemented *Neurog1*^*+/−*^ inner ears.

## Data Availability

Data sharing is not applicable to this article as no datasets were generated or analyzed during the current study.

## Abbreviations

AA: Anterior ampulla
AAALAC: American association for accreditation of laboratory animal care
ATOH1: Atonal homolog 1
ASCC: Anterior semicircular canal
BABB: Benzyl alcohol:benzyl benzoate
BC: Blastocyst complementation
E: Embryonic day
EDTA: Ethylenediaminetetraacetic acid
ESCs: Embryonic stem cells
DAPI: 4′,6-Diamidino-2-phenylindole
DPI: Dot per inch (which reflects pixels per inch)
FGFR2: Fibroblast growth factor receptor 2
FIJI: Fiji Is Just ImageJ
GFP: Green fluorescent protein
hESCs: Human embryonic stem cells
HC: Hair cell
HCl: Hydrogen chloride
HCG: Human chorionic gonadotropin
hiPSCs: Human-induced pluripotent cells
HTF/mHTF: Human tubal fluid and modified human tubal fluid
IACUC: Institute of animal care use committee
*i.p.*: Intraperitoneally
iPSCs: Induced pluripotent stem cell
IHC: Immunohistochemistry
LA: Lateral ampulla
LIF: Leica image file format
LSCC: Lateral semicircular canal
MHC: Major histocompatibility complex
MITF: Melanocyte inducing transcription factor
MYO6: Unconventional myosin VI
*Neurog1*: *Neurogenin1*
N-Myc: *N*-Myc proto-oncogene protein
P: Postnatal day
PA: Posterior ampulla
PBS: Phosphate-buffered saline
PBST: Phosphate-buffered saline with 0.1% Triton X-100
PCR: Polymerase chain reaction
PD: Parkinson’s disease
PDX1: Pancreatic and duodenal homeobox1
PFA: Paraformaldehyde
PSCC: Posterior semicircular canal
RAR: Research animal resources
RC: Rosenthal’s Canal with SGNs
SAC: Saccule
SALL1: Sal-like like protein 1
SGNs: Spiral ganglion neurons, the auditory nerve
SPF: Specific pathogen-free
SNHL: Sensorineural hearing loss
sTSLIM: Scanning thin sheet laser image microscope
TFM: Tissue freezing medium
TUJ1: Class III beta-tubulin
Ut: Utricle
VG: Vestibular ganglion
