# Supplementary material for: Generation of inner ear sensory neurons using blastocyst complementation in a Neurog1+/−−deficient mouse
Source: Stem Cell Res Ther. 2021 Feb 12;12:122. doi: 10.1186/s13287-021-02184-1 (PMC7881691; doi:10.1186/s13287-021-02184-1)
Supplement: Supplementary file 1 — Additional file 1: Supplemental Figure 1 Representative gel images of genotyping for a litter from the Neurog1 colony. Top gel is the mutant genotyping, and the bottom gel is the wild type genotyping for the same samples. Wild type (lane 1): 198 bp. Heterozygote (lanes 2-4,6-13): 198 bp and ~ 500 bp. Mutant (lane 5): ~ 500 bp. Heterozygote positive controls (lanes 11-13) were always run in triplicate due to occasional variability in their result. All genotyping was run 3 times for each sample to confirm genotype. Supplemental Figure 2. Representative examples of GFP-expressing induced pluripotent stem cell (iPSC) colonies. (A) GFP expression. (B) Phase Contrast. Note that all of the iPSCs are GFP-labeled. Scale Bar: 100 μm. Supplemental Figure 3 2D optical section through a wild type cochlea using sTSLIM. Bracket indicates the sensory cells of the organ of Corti. Note the high degree of resolution throughout the image, in particular the cell bodies in the SGN. Scale Bar: 100 μm. oC: organ of Corti; SGN: Spiral ganglion neurons. [file 13287_2021_2184_MOESM1_ESM.docx]

**Supplemental material**

**Supplemental Fig 1.**

**
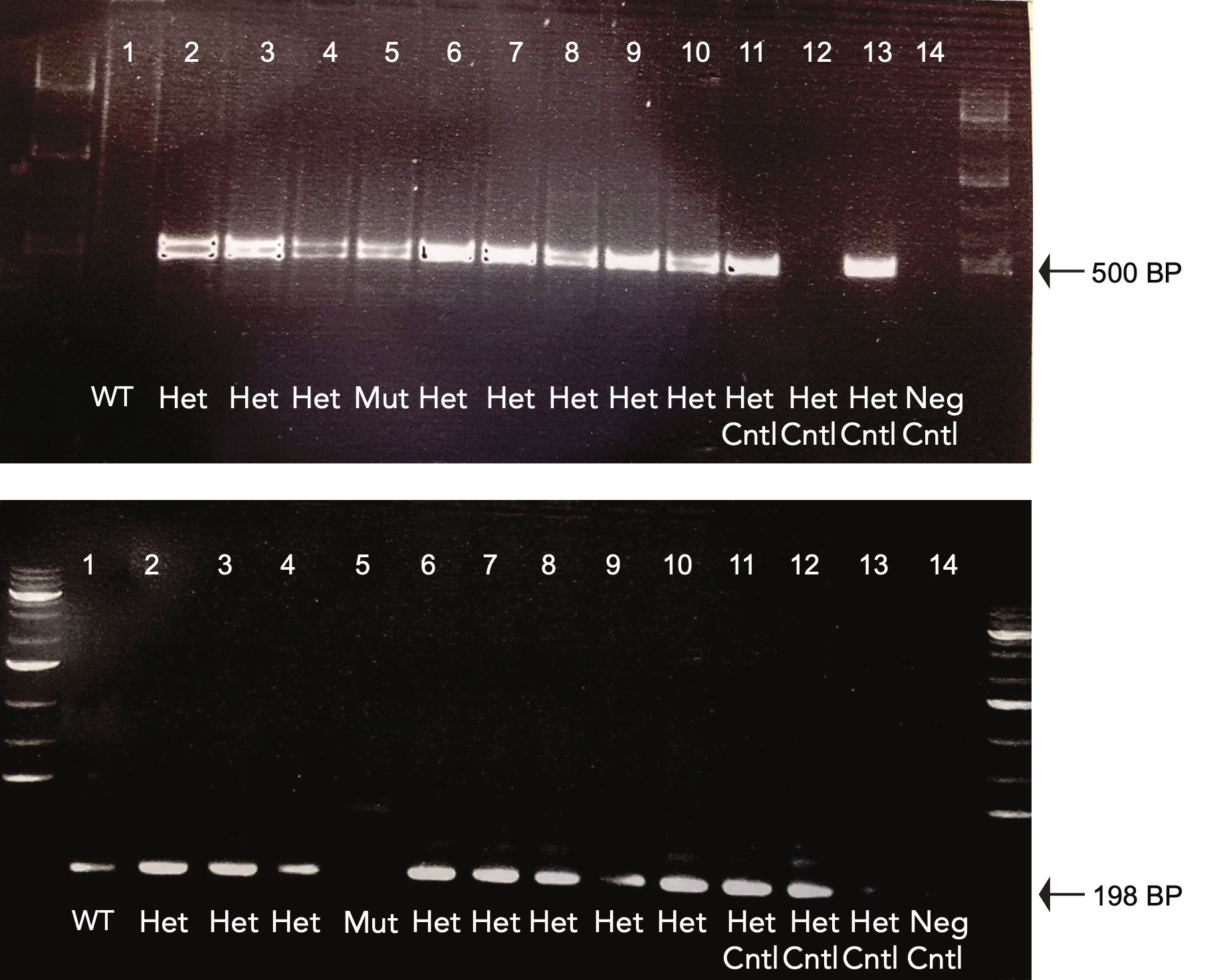
**

**Supplemental Fig 1.**  Representative gel images of genotyping for a litter from the *Neurog1* colony. Top gel is the mutant genotyping, and the bottom gel is the wild type genotyping for the same samples. Wild type (lane 1): 198 bp. Heterozygote (lanes 2-4,6-13): 198 bp and ~ 500 bp. Mutant (lane 5): ~ 500 bp. Heterozygote positive controls (lanes 11-13) were always run in triplicate due to occasional variability in their result. All genotyping was run 3 times for each sample to confirm genotype.

**Supplemental Fig 2.**

**
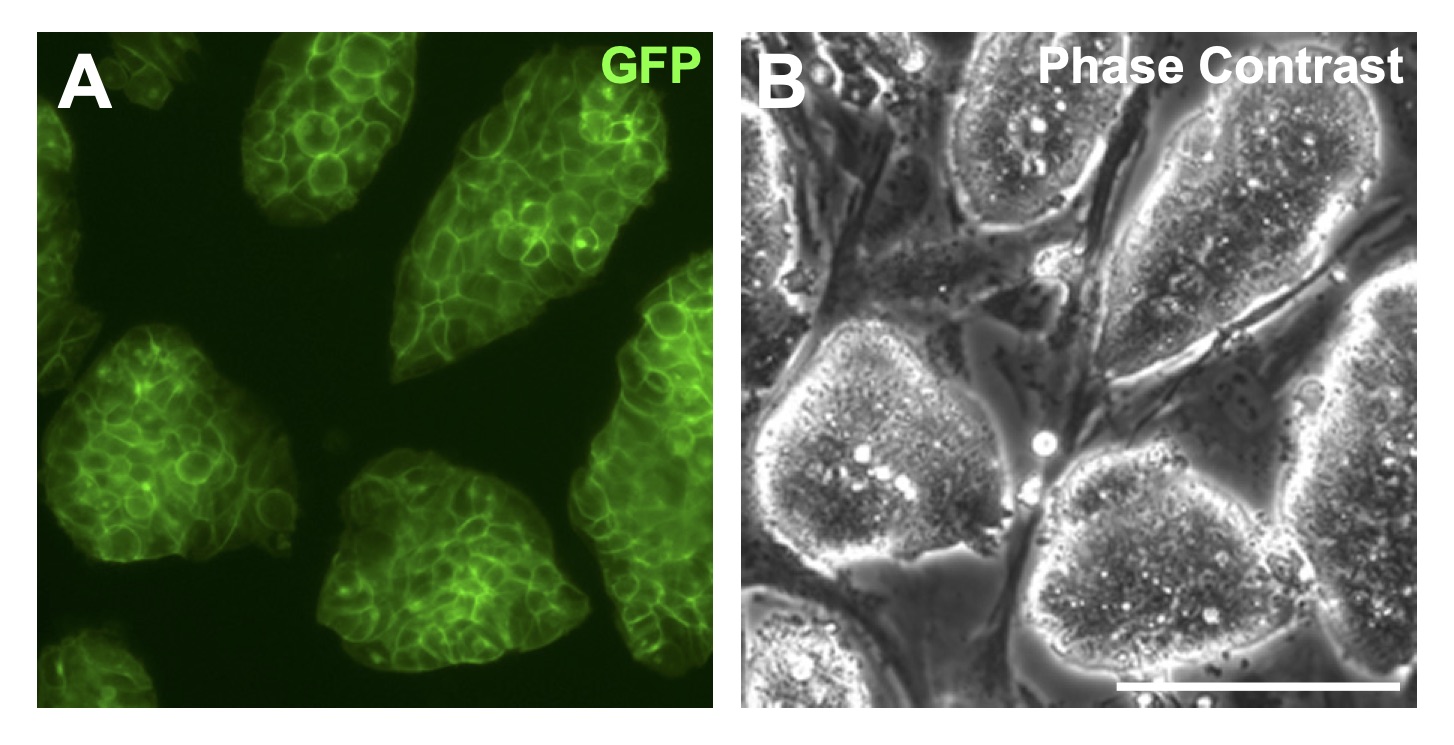
**

**Supplemental Fig 2.**  Representative examples of GFP-expressing induced pluripotent stem cell (iPSC) colonies. (A) GFP expression. (B) Phase Contrast. Note that all of the iPSCs are GFP-labeled. Scale Bar: 100 µm.

**
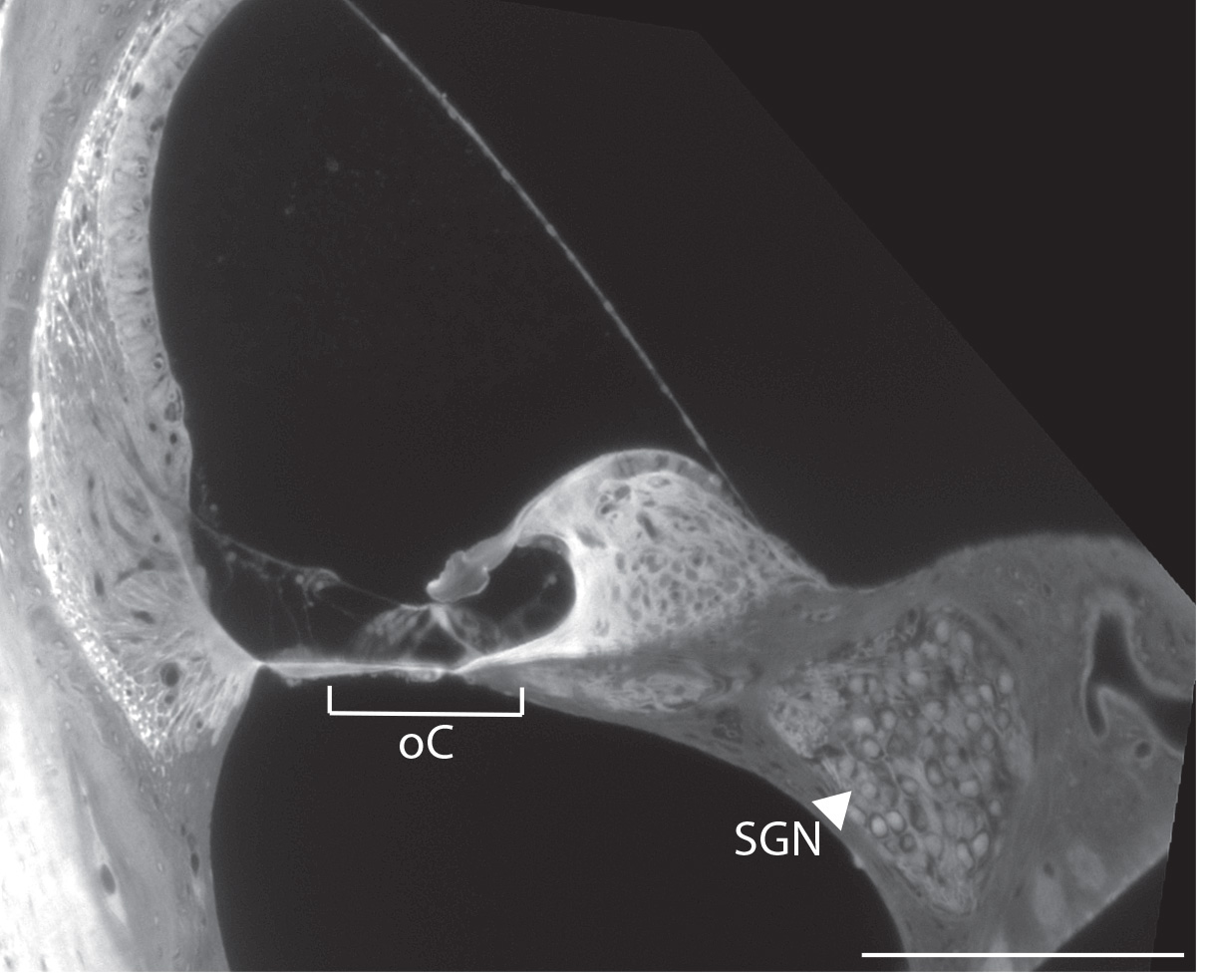
**

**Supplemental Fig. 3.**  2D optical section through a wild type cochlea using sTSLIM. Bracket indicates the sensory cells of the organ of Corti. Note the high degree of resolution throughout the image, in particular the cell bodies in the SGN. Scale Bar: 100 µm. oC: organ of Corti; SGN: Spiral ganglion neurons
